# Effectiveness of predator satiation in masting oaks is negatively affected by conspecific density

**DOI:** 10.1007/s00442-018-4069-7

**Published:** 2018-01-30

**Authors:** Michał Bogdziewicz, Josep M. Espelta, Alberto Muñoz, Jose M. Aparicio, Raul Bonal

**Affiliations:** 1 Department of Systematic Zoology, Faculty of Biology, Adam Mickiewicz University, Umultowska 89, 61-614 Poznań, Poland; 2 CREAF, Cerdanyola del Valles, 08193 Catalonia, Spain; 3 Departamento de Didáctica de la Ciencias Experimentales, Facultad de Educación, Universidad Complutense de Madrid, Madrid, Spain; 4 Grupo de Investigación de la Biodiversidad Genética y Cultural, Instituto de Investigación en Recursos Cinegéticos (CSIC-UCLM-JCCM), Ciudad Real, Spain; 5 Forest Research Group, INDEHESA, University of Extremadura, Plasencia, Spain; 6 DITEG Research Group, University of Castilla-La Mancha, Toledo, Spain

**Keywords:** *Curculio*, Density-dependent predation, Plant reproduction, Predator functional response, Seed predation

## Abstract

Variation in seed availability shapes plant communities, and is strongly affected by seed predation. In some plant species, temporal variation in seed production is especially high and synchronized over large areas, which is called ‘mast seeding’. One selective advantage of this phenomenon is predator satiation which posits that masting helps plants escape seed predation through starvation of predators in lean years, and satiation in mast years. However, even though seed predation can be predicted to have a strong spatial component and depend on plant densities, whether the effectiveness of predator satiation in masting plants changes according to the Janzen-Connell effect has been barely investigated. We studied, over an 8-year period, the seed production, the spatiotemporal patters of weevil seed predation, and the abundance of adult weevils in a holm oak (*Quercus ilex*) population that consists of trees interspersed at patches covering a continuum of conspecific density. Isolated oaks effectively satiate predators, but this is trumped by increasing conspecific plant density. Lack of predator satiation in trees growing in dense patches was caused by re-distribution of insects among plants that likely attenuated them against food shortage in lean years, and changed the type of weevil functional response from type II in isolated trees to type III in trees growing in dense patches. This study provides the first empirical evaluation of the notion that masting and predator satiation should be more important in populations that start to dominate their communities, and is consistent with the observation that masting is less frequent and less intense in diverse forests.

**Electronic supplementary material:**

The online version of this article (10.1007/s00442-018-4069-7) contains supplementary material, which is available to authorized users.

## Introduction

In probably most plant populations, production of seeds varies widely between years (Herrera et al. 1998). Fluctuations in seed production shape plant communities through their effects on competition, population dynamics and seed predator numbers (Crawley and Long 1995; Ostfeld and Keesing 2000; Curran and Webb 2000; Wright et al. 2005; Bogdziewicz et al. 2016). In some plant species, the high annual variation in reproductive output is synchronized over large areas, which is called ‘mast seeding’ or ‘masting’ (Kelly 1994). This variation is thought to render a fitness benefit to plants through enhanced pollination efficiency (Rapp et al. 2013; Moreira et al. 2014), higher seed dispersal by animals (Vander Wall 2002; Pesendorfer et al. 2016), and satiation of seed predators (Kelly and Sork 2002; Bogdziewicz et al. 2017a). The predator satiation hypothesis (PSH) is the most supported one in this regard, and posits that masting helps plants to escape seed predation through starvation of predators in lean years, and satiation of seed predators in bumper years, ultimately resulting in positive density dependence of seed survival (Kelly 1994; Kelly and Sork 2002; Wright et al. 2005; Pearse et al. 2016). However, besides effects mediated by temporal variability in seed production, seed predation has also a strong spatial component (Janzen 1970; Wright 2002; Comita et al. 2014). It has been long hypothesized that predator satiation effectiveness is attenuated by the focal plant density (Janzen 1971, 1978; Curran and Webb 2000; Kelly and Sork 2002; Koenig et al. 2003), but the extent to which this is true remains barely explored (but see Xiao et al. 2016).

The PSH has two components. First, satiation is represented by a predator’s type II or III functional response, i.e. there is a decreasing proportion of predated seeds with increasing seed density (Kelly et al. 2000; Fletcher et al. 2010; Żywiec et al. 2013; Linhart et al. 2014; Moreira et al. 2016, 2017). In the type II functional response, the predation rate decreases with increasing seed availability (Holling 1965). In the type III, the predation rate increases up to some point, as predators switch to increasingly available prey, and the rate possibly decreases afterwards as predators become satiated (Holling 1965). The second component of predator satiation in masting is starvation of seed predators in low seed years, i.e. a numerical response that should help plants escape predation in mast years as it is easier to satiate a lower number of enemies (Silvertown 1980; Kelly and Sullivan 1997; Espelta et al. 2008; Xiao et al. 2013; Zwolak et al. 2016). Although never empirically evaluated, we argue that seed escape through predator satiation might diminish with increasing focal plant density.

The Janzen-Connell effect (JC) proposes that seed or seedling survival is negatively related to conspecific population density due to the aggregation of specialist enemies in patches with high densities of seeds or adults of their host plant species (Janzen 1970; Connell 1971; Wright 2002). The JC effect and PSH have been so far studied separately (but see Xiao et al. 2016). However, predator satiation can be disrupted if predators can move between plants and relocate predation pressure across different conspecifics within the same patch (Platt et al. 1974; Ims 1990; Wyatt and Silman 2004). In this context, the negative consequences of lean years on seed predator numbers would not be so strong, as they could always find some plants with a good seed production to feed on (Ims 1990). Therefore, we could expect the numerical component of PSH to be less efficient in dense plant patches. Furthermore, for seed predators with relatively low dispersal abilities, like numerous granivorous insects, between-tree migration is more likely in cases of more closely spaced plants than in isolated trees (Platt et al. 1974; Kelly and Sork 2002; Wright et al. 2005, Moreira et al. 2017). The functional responses of predators to prey availability are highly contingent upon predator traits, including mobility (Moreira et al. 2017). Notably, less mobile predators (e.g. many seed-feeding insects) frequently exhibit the type II functional response, while mobile predators the type III one (Żywiec et al. 2013; Linhart et al. 2014; Moreira et al. 2017). Close proximity of trees in dense conspecific patches may allow less mobile predators to move between plants, changing the expected type of the functional response from II to III. Depending on whether or not the type III functional response includes a decrease in predation rate, this could further decrease the effectiveness of predator satiation at times of masting.

We evaluated spatiotemporal factors shaping seed predation in our study system: evergreen Mediterranean holm oak (*Quercus ilex*) that shows typical masting behaviour (Pérez-Ramos et al. 2010; Fernández-Martínez et al. 2015; Bogdziewicz et al. 2017b), interacting with seed-feeding chestnut weevil (*Curculio elephas*). We monitored acorn production and predation over 8 years (2008–2015) in 24 oak trees interspersed at patches of different conspecific density. We also monitored weevil imago abundance over corresponding six seasons (2008–2013). Chestnut weevils are specialist seed predators with low mobility (Venner et al. 2011; Pelisson et al. 2013a, b), and are the most important predators of oak holm acorns capable of destroying entire crops of individual trees (Bonal et al. 2007; Espelta et al. 2008, 2017). We evaluated the types of functional response of weevils to acorn availability, and whether these differ across focal oak density. We predicted that weevils should exhibit the type II functional response in isolated trees, due to their relatively low mobility (Moreira et al. 2017). In contrast, in trees growing in dense patches, low mobility of weevils should no longer be a constraint, allowing migration between trees and aggregation in resource-rich trees, i.e. the type III functional response. We also assessed the numerical response of imago weevils to temporal variation in acorn production in relation to oak densities, and its consequences for acorn predation. We expected that less variable food provision in trees growing in dense patches should decrease the efficiency of the numerical component of the PSH, i.e. acorn predation and weevil imago abundance should strongly depend on the size of the previous year’s crop in isolated oaks.

## Materials and methods

### Study site and species

The study area is located in Huecas, Toledo Province, Central Spain (39°59′N, 4°13′W). It is dominated by extensive cropland, mainly barley (*Hordeum vulgare*) and wheat (*Triticum* spp.), with holm oaks *Quercus ilex* interspersed within the agricultural matrix (Picture 1). Some of the holm oaks are isolated, and some of them form small groups or forest stands; the mean distance among sampled trees (*n* = 24) is 1907 m (range from 6 m to 4033 m) (Bonal et al. 2012; Ortego et al. 2014). The holm oak is the most widespread tree species of the Iberian Peninsula, and these fragmented populations in Central Spain are the remnants of former forests that were cleared for centuries along with agricultural development (Ortego et al. 2014). Holm oaks flower in spring and acorns grow and ripen in the same year; the acorns are dropped in autumn and winter. Crop sizes are subjected to strong inter-annual fluctuations (Espelta et al. 2008, Bogdziewicz et al. 2017b) and can be very high in certain years; a single tree may produce over 35,000 acorns (Bonal et al. 2007).

**Picture 1:**
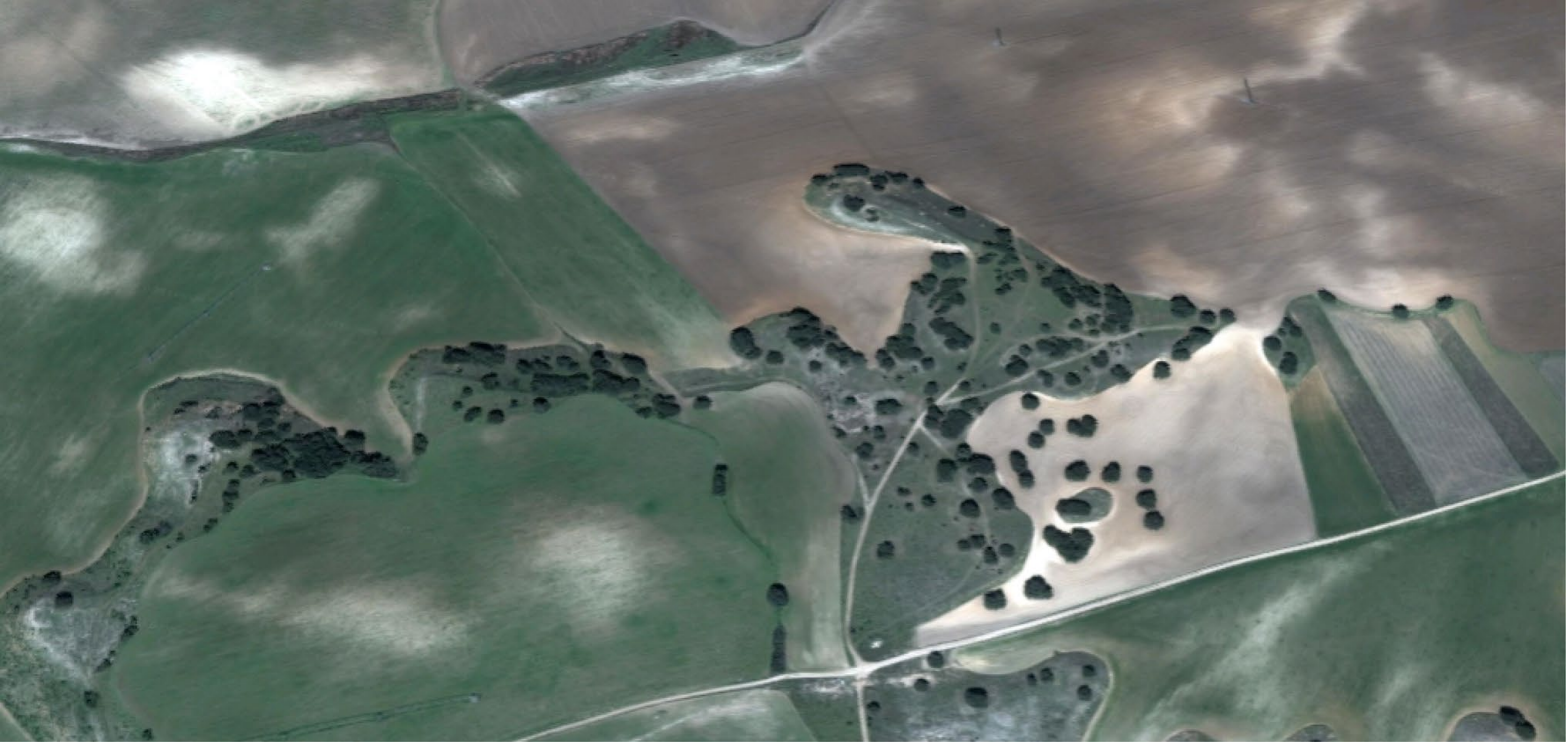
Study area. Monitored holm oaks interspersed within the agricultural matrix, Huecas, Spain

*Curculio elephas* is a specialist insect that feeds on acorns and chestnuts; in Central Spain, where there are no chestnuts, it is the main pre-dispersal predator of holm oak acorns (Bonal et al. 2007, 2012). Adult females perforate a tiny hole in the seed coat with their long rostrum and oviposit into the acorns using their oviscapt (Desouhant et al. 2000). Infested acorns are dropped prematurely and larvae grow within them feeding on the cotyledons until completing development, when they drill an exit hole through the seed coat to leave the acorn and bury themselves underground. Larvae overwinter within earth cells and may experience extended diapause: i.e. most adult weevils emerge after 1 year, but some may go through a longer diapause and spend 2 or even 3 years underground [respectively 66, 30 and 4% according to Venner et al. (2011)]. After emergence, *C. elephas* adults climb the trunk of the oak under which they emerged up to the canopy where they mate and females oviposit (Bonal et al. 2012). The focal weevil shows poor dispersal ability, i.e. 600 m according to Pelisson et al. (2013b) and restricted gene flow after 300 m (H. Ruíz-Carbayo, unpublished data).

### Acorn crop and weevil predation

We monitored 24 randomly chosen trees from 2008 till 2015. We estimated the acorn crop of individual trees by placing seed traps (buckets of 0.12 m^2^) under the tree canopies. Seed traps were sampled periodically and, after the first acorns were found, traps were checked every 10 days until acorn fall ceased. The number of traps differed between trees to cover the same proportion of canopy surface for all of them (between 1.5 and 2%). We calculated tree canopy surface on the basis of three measures of the diameter of their canopy, considering trees to be roughly circular. After every sampling, we took collected acorns to the laboratory, where we classified them as ‘infested’ or ‘sound’. Acorns presenting larval exit holes were directly classified as infested, whereas those with only oviposition scars were placed in individual plastic vials to record insect emergence (Bonal and Muñoz 2009). One month after the last larva had emerged, we opened all acorns (initially classified as sound or infested) to confirm classification. Besides *Curculio* spp. weevils there are other acorn borer insects, mainly *Cydia* spp. (Lepidoptera) caterpillars, although previous studies in Central Spain have found low prevalence (below 10%) (Bonal et al. 2007) and were thus not considered in this study. We calculated the total number of grown acorns (infested plus sound ones) produced by each tree per square metre and in total (calculated as the production per square metre by the canopy surface). These acorn crop estimates are accurate, as differences between the seed traps of each tree are very small compared with between-tree variability (Bonal et al. 2007).

### Index of weevil imago abundance

To estimate the abundance of adult weevils per tree from 2008 till 2013 we used the standard method (described in Bonal et al. 2012) of trapping adult weevils using emergence traps. Each trap consisted of a mosquito net attached to the tree trunk with an inverted cone with a closed bottle on the top. The weevils climbed up the trunk after leaving their underground cells and marched into the net, which led them directly to the top bottle, where they were trapped. The traps partially covered the tree trunk, and the number on each tree ranged from one to three (median = 2), in proportion to the tree canopy. Traps were attached to the trunk very close to the ground. This ensured that the captured adults were individuals that had pupated underground beneath the tree canopy and had climbed the tree trunk after adult emergence. Emigration from tree to tree is highly improbable, except in dense forest areas, owing to the lower dispersal ability of this species (Venner et al. 2011; Pelisson et al. 2013a, Pelisson et al. 2013b). Moreover, the potential immigrants are more likely to come from other trees by flying or marching onto the canopies in dense forests (Bonal et al. 2012, 2015). Notice that this method provides an estimate of the population size of adult weevils but it cannot be considered an exhaustive census, as the traps only partially covered the tree trunk. In fact, our aim was not to trap all weevils, as we wanted most of the weevils to climb up to the tree canopy so as not to interfere with the infestation rates.

### Measures of connectivity

In the analyses, we used the connectivity index (Puerta-Pinero et al. 2012; Ruiz-Carbayo et al. 2017) as an index of spatial distribution of trees within the landscape. This index measures within one value the connectivity of a focal tree with the rest of the surrounding conspecifics, encompassing both density of plants and spatial patterns of plant location in the landscape (Hanski 1999; Puerta-Pinero et al. 2012). We geolocated the study oaks in the field using a global positioning system, georeferenced all holm oak trees in the study area, and used orthoimages to determine tree crown perimeters and then the tree crown area for selected oaks, using Miramon geographic information system tools (Ruiz-Carbayo et al. 2017). We estimated the connectivity for each study tree (*C*_i_) using a modification of Hanski’s connectivity index (Hanski 1999): $C_{i} = \sum {\left( {I_{j} \times e^{{ - d_{i,j} }} } \right)} ,$ where *I*_*j*_ corresponds to pixels of holm oak tree or patch different from those of the study tree *i*, and *d*_*i,j*_ to its distance to the edge of the study tree.

### Data analysis

All the analyses were done at the individual-tree level. All sampled trees in the population were categorized based on their connectivity values. The categorization was based on the dispersal abilities reported for *C. elephas* (600 m) (Pelisson et al. 2013b). In the ‘isolated’ category, trees were spaced at almost three times the dispersal distance of 600 m (mean 1726 m), while trees in dense patches were spaced at one-third that dispersal distance (mean 220 m). The average connectivity in the isolated category equalled 63.48 (range 7–164, *n* = 15 trees), and in dense patches 641 (453–803, *n* = 9 trees). We used oak patch density as a categorical (isolated trees vs. trees growing in dense oak patches) instead of a continuous variable, in order to aid interpretation. Nonetheless, the results were qualitatively the same using either categorical or continuous conspecific connectivity as an independent variable, and the latter analysis is presented in the Online Supplement (Table S1).

We calculated masting metrics for the acorn production dataset, which included average seed production, population-level (CV_p_) and individual-level (CV_i_) coefficients of variation (SD/mean), and synchrony of seed production among trees (mean Pearson cross-correlation) (Koenig et al. 2003; Crone et al. 2011). We calculated CV_p_ based on annual means (across trees). We calculated the metrics for the general population, as well as for isolated and dense patches separately. We also calculated mean index of weevil imago abundance per tree, and mean index of imago weevil abundance per tree in relation to acorn availability (no. of weevils per acorn).

We assessed the functional response of weevil seed predation to acorn abundance, and whether this differed among trees growing in patches with different oak densities (isolated vs. dense), using generalised linear mixed models (GLMMs) implemented via the glmmADMB package in R (Fournier et al. 2012). We used tree identifier (ID) as a random effect, a binomial family error term, and logit link. We did not include year as a random effect as it would have restricted the assessment of the functional response to variation in available acorns within each year, while the among-year variation is relevant for the PSH in masting (Fletcher et al. 2010). We used acorn predation rate by weevils at tree-level as binomial response, and constructed candidate models that included different combinations of the linear and quadratic term of acorn crop, and their interaction with oak density category as fixed effects. The type I functional response is described by non-significant linear and quadratic coefficients of acorn abundance, the type II response is indicated by a significant negative linear coefficient, and the type III response is indicated by a significant positive linear coefficient and a negative quadratic coefficient.

Next, we modelled the tree-level predation rate on the number of imago trapped at each particular tree. This was done in order to test whether the differences in the functional response of weevils to acorn abundance in isolated trees vs. those growing in dense areas arise as a consequence of imago weevil migration between trees in the latter. Here, we constructed similar binomial GLMMs as above, but we included log-transformed mean index imago number emerged under the tree in a particular year, stand density category, and interaction term as fixed effects. If the level of migration between trees differs among trees growing in different densities, e.g. is higher in trees growing in dense patches, we expected no relationship between the index of imago abundance emerged under a tree and predation rate in trees growing in dense stands. In contrast, in isolated trees the migration should be virtually non-existent [according to the mean distance among trees and the dispersal distance for *C. elephas* (Pelisson et al. 2013a, b)]. Thus, the seed predation rate should be linked to the abundance of imago emerged under the tree.

**Fig. 1:**
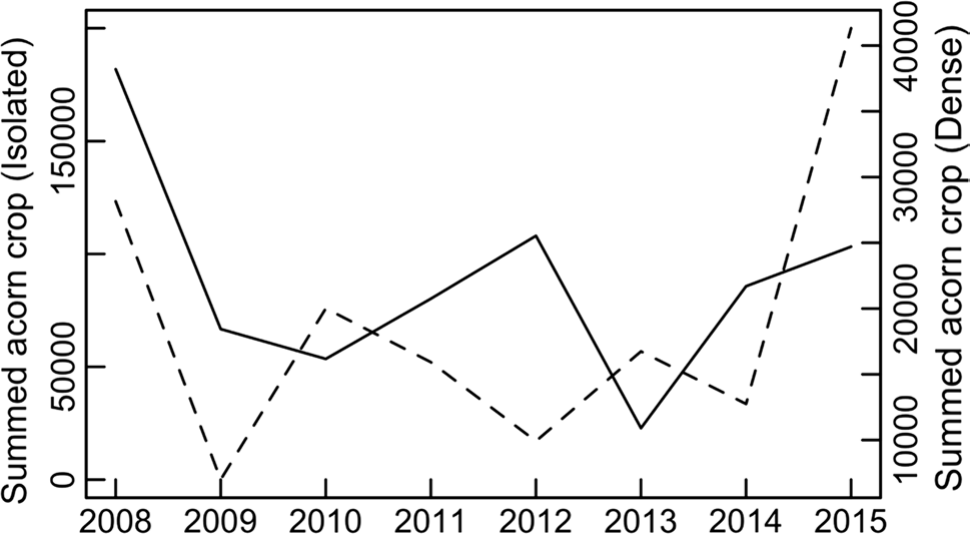
Summed yearly acorn production of holm oaks.* Solid line* represents patters of seed production of all trees classified as isolated, while* dashed line* represents all trees classified as growing in dense patches. Note the different scales

We assessed whether patch density affects the numerical component of the PSH using two analyses. In the first, we modelled whether individual-tree level acorn predation rate is affected by the number of acorns produced by the tree in the last 2 years. Here, we constructed similar binomial GLMMs as above, but included summed acorn production in the last 2 years, stand density category, and interaction term as response variables. We included summed number of acorns in the previous 2 years to account for the variable diapause abilities of *C. elephas* (Venner et al. 2011) that comprise over 90% of the weevil population in our study area (Bonal et al. 2015). Models that included only crop of the previous year failed to predict imago abundance (Online Supplement, Fig. 1S). In the next test, we used the number of imago weevil trapped at a particular tree as the response, and log-transformed overall acorn crop size of the tree in the previous 2 years, patch type category, and the interaction term as fixed effects. Here, we used zero-inflated, negative binomial, GLMMs with tree ID and year as random effects. In this analysis, year was included as a random effect to account for the effects of environmental stochasticity on weevil populations (Bonal et al. 2015; Espelta et al. 2017). We used a negative binomial, rather than Poisson models, as the former fitted the data better [comparison of full models, difference in Akaike information criterion adjusted for small sample sizes (ΔAICc) > 50]. We used number of traps attached per tree as an offset.

We followed an information-theoretic approach to identify the most parsimonious models for each analysis (Burnham and Anderson 2002). In each analysis, we constructed sets of models based on the full model, and compared models within a given set based on their values of AICc. The model with the lowest AICc within the set was considered the best, given the data (Burnham and Anderson 2002), but we discarded models with uninformative parameters that were more complex versions of a competitive model with fewer parameters whose ∆AICc < 2 (Arnold 2010). In all the top models, we checked for the spatial autocorrelation of residuals by plotting them against geographical coordinates, and by drawing variograms. No spatial autocorrelation was detected in either model. Detailed information on the sample size used in each analysis is provided in the figure captions.

## Results

Holm oaks at our study site showed relatively low population-level variation, and moderate individual-level variation in seed production (Fig. 1; Table 1). The mean crop size was twice as high in trees growing in isolation as in trees growing in high density patches. The index of imago abundance was similar in both density categories, but imago weevil abundance relative to acorn availably was twice as high in dense patches (Table 1).

**Table 1:** Masting metrics for holm oak (*Quercus ilex*) population, and weevil (*Curculio elephas*) abundance at our study site; SDs are given* in parentheses*

	Isolated	Dense	General population
Population-level coefficient of variation	0.55	0.49	0.43
Individual-level coefficient of variation	1.00 (0.28)	0.91 (0.36)	0.96 (0.31)
Synchrony (*r*)	0.14 (0.36)	0.37 (0.29)	0.13 (0.37)
Mean crop per tree	5850 (6501)	2108 (1206)	4447 (5446)
Mean index of weevil imago abundance per tree	1.37 (2.30)	1.52 (1.80)	1.43 (2.08)
Mean index of weevil imago per acorn	119 × 10^−5^	278 × 10^−5^	138 × 10^−5^

**Fig. 2:**
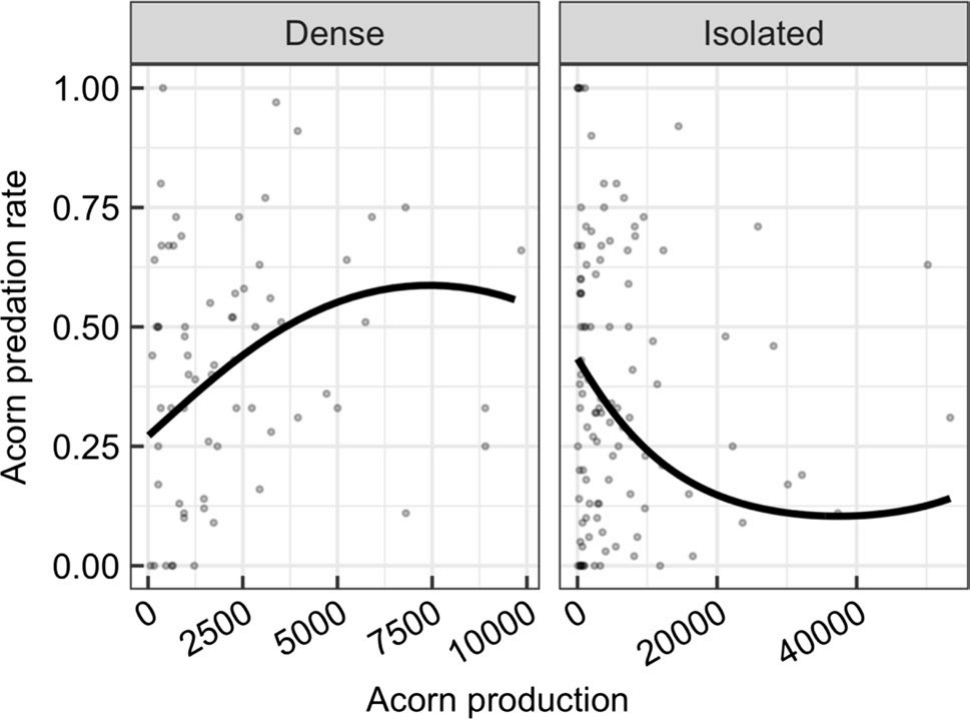
Relationship between tree-level acorn production and tree-level predation rate in dense and isolated patches of holm oak.* Dots* represent data points, i.e. tree by year observations, *n* = 67 for dense, *n* = 109 for isolated patches (some trees failed to produce acorns in some years, which precluded calculating predation rate).* Trend lines* are based on a generalised linear mixed model (GLMM) fitted to the data (see “Materials and methods” section for details)

The top model assessing the functional response of weevil predation rate to acorn availability included interaction terms between crop size and stand density category, and between quadratic term of crop size and stand density category (Table 2A). The model revealed a negative linear relationship (*z* = − 12.54, *p* < 0.001), and a positive quadratic (*z* = 9.90, *p* < 0.001) relationship between crop size and predation rate in isolated trees (Fig. 2a). The non-linear decrease in predation rate with increasing crop size implies a type II functional response in isolated stands (Fig. 2). In contrast, in trees growing in dense patches, the linear coefficient was positive (*z* = 4.62, *p* < 0.001), while the quadratic term was negative (*z* = − 3.01, *p* = 0.002), indicating a type III functional response. However, predation only increased up to a certain level, but did not start to decrease, indicating lower effectiveness of predator satiation in these patches (Fig. 2b).

**Table 2:** Model selection table

Model	*df*	Log likelihood	AIC	ΔAICc	*w*_i_
(A) Functional response of weevil predation to acorn abundance in relation to oak density					
Crop size × stand density + (crop size)^2^ × stand density	7	− 1363.4	2741.5	0	1
Crop size × stand density	5	− 1418.0	2846.4	104.91	0
Crop size + stand density	4	− 1447.1	2902.5	160.99	0
Crop size	3	− 1449.6	2902.5	164.01	0
Stand density	3	− 1498.2	3002.7	261.21	0
Null model (intercept only)	2	− 1503.7	3011.6	270.16	0
(B) Relationship between acorn predation rate and number of imago emerged from under a tree in relation to oak patch density					
Log-imago abundance × stand density	5	− 807.5	1625.4	0	0.97
Log-imago abundance + stand density	4	− 812.2	1632.8	7.28	0.03
Stand density	3	− 822.7	1651.7	26.15	0
Null model (intercept only)	2	− 825.7	1655.6	30.09	0
(C) Relationship between acorn predation rate and summed crop of 2 previous years in relation to oak patch density					
Crop size × stand density	5	− 1055.9	2122.4	0	0.77
Crop size + stand density	4	− 1058.3	2124.9	2.52	0.22
Crop size	3	− 1062.6	2131.6	9.18	0.01
Stand density	3	− 1129.8	2265.8	143.46	0
Null model (intercept only)	2	− 1132.2	2268.6	146.17	0
(D) Relationship between imago abundance and summed crop of 2 previous years in relation to oak patch density					
Crop size × stand density	8	− 188.2	394.3	0	0.98
Crop size + stand density	7	− 193.6	402.8	8.48	0.02
Crop size	6	− 195.9	404.9	10.61	0
Null model (intercept only)	5	− 199.3	409.4	15.17	0
Stand density	6	− 198.6	410.3	16.00	0

In the presented models oak density is included as a categorical variable (isolated vs. dense patches) to enhance interpretation and visualisation of the results. The results are qualitatively the same if density is used as a continuous variable (see Online Supplement 1, Table S1). Models are ranked according to Akaike’s information criterion adjusted for small sample size (*AICc*)

*ΔAICc* AICc_i_-minimum AICc, *w*_*i*_ model weight, *times symbol* interaction term

**Fig. 3:**
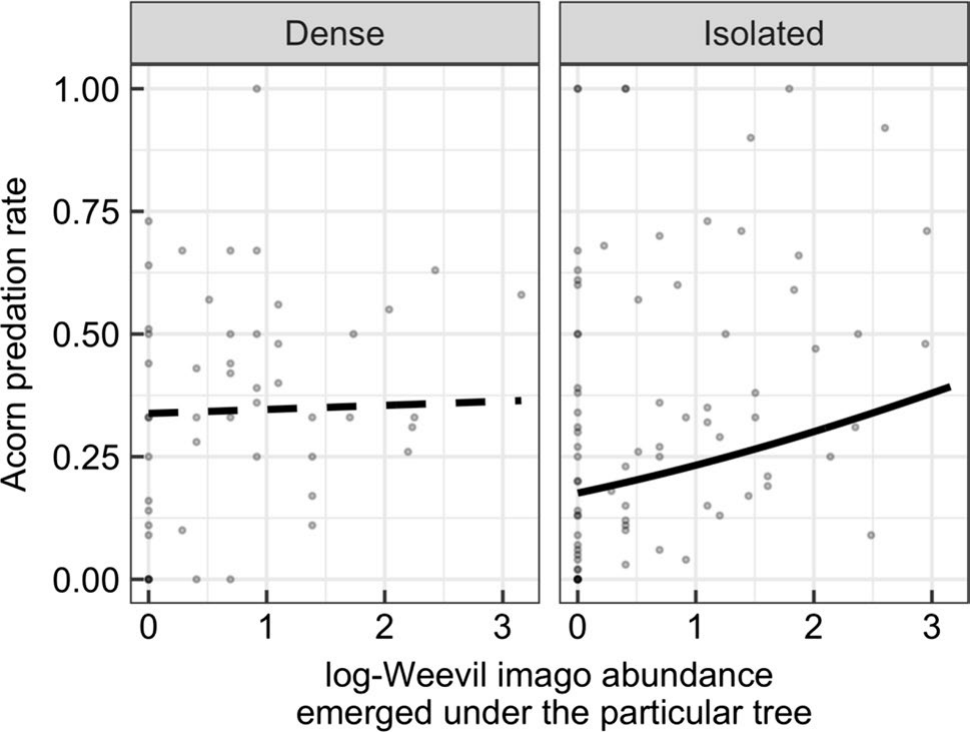
Relationship between the index of number of imagos emerged under trees in a particular year and acorn predation rate of the same trees in dense and isolated patches.* Dots* represent data points, i.e. tree by year observations, *n* = 50 for dense and *n* = 83 for isolated trees (some trees failed to produce acorns in some years, and imago abundance was monitored for 2 fewer years than for acorn production, see “Materials and methods” section for details).* Trend lines* are based on GLMM fitted to the data (see “Materials and methods” section for details). *Dashed line* shows a non-significant relationship

The top model describing the relationship between predation rate and number of emerged weevil imago under a particular tree in the same year, included the interaction term between imago abundance and stand density category (Table 2B). The predation rate was positively related to imago abundance in isolated trees (*z* = 5.56, *p* < 0.001; Fig. 3), but this was not the case in trees in dense patches (*z* = 0.45, *p* = 0.65; Fig. 3).

**Fig. 4:**
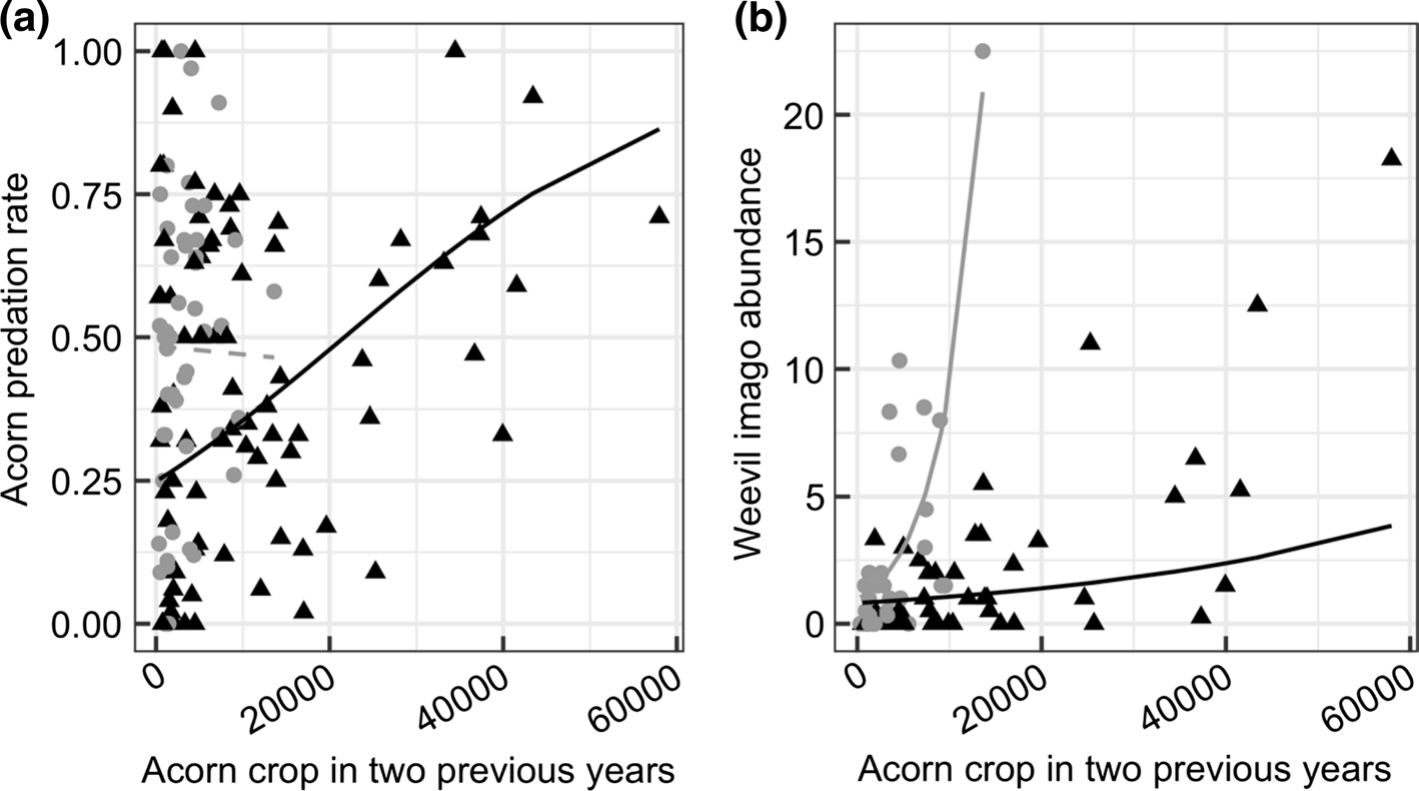
Relationship between tree-level summed acorn production in the 2 previous years and **a** tree-level acorn predation rate, and **b** weevil imago abundance per tree in dense (grey circles) and isolated (black triangles) patches. Dots represent data points, i.e. tree by year observations, *n* = 36 for dense and *n* = 60 for isolated trees (this analysis required aligning years, thus it was based on data of 2 fewer years). Trend lines are based on GLMM fitted to the data (see “Materials and methods” for details). In **b** the prediction is given per two weevil traps, and dots are the average number of weevil imagos trapped per tree in each year. Dashed line shows a non-significant relationship

In models relating predation rate to summed acorn crop in the last 2 years, the top model included the interaction term between acorn crop size and stand density category (Table 2C). The model revealed dependence of predation rate on summed acorn crop in the previous 2 years in isolated trees (*z* = 11.50, *p* < 0.001; Fig. 4a), but not in trees in dense patches (*z* = − 0.23, *p* = 0.83). Similarly, the top model describing the relationship between the index of the imago abundance and summed crop of the 2 previous years included the interaction between crop size and stand density category (Table 2D). Here, the effect of crop size was positive in both density categories (trees in dense patches, *z* = 4.05, *p* < 0.001; isolated trees, *z* = 2.62, *p* = 0.008), although the slope was higher for trees growing in dense patches (*z* = 3.44, *p* < 0.001; Fig. 4b). Note, however, that the interaction was not significant when oak connectivity (index of spatial distribution of trees within the landscape) was included as a continuous variable (cf. Table S1).

Generally, predation rate was higher in trees growing in dense oak stands than in isolated trees (mean % ± SD, isolated, 0.38 ± 0.28; dense, 0.42 ± 0.25; *z* = 4.07, *p* = 0.021), while weevil imago abundance was similar across densities (*z* = 0.29, *p* = 0.76; Table 1).

## Discussion

Both temporal (PSH) and spatial (JC effect) variation in seed production shaped pre-dispersal seed predation in masting trees, i.e. the effectiveness of predator satiation was strongly dependent on the density of focal trees. In isolated tree patches, the type II functional response indicated the satiation effect. In contrast, the close proximity of oaks in dense patches allowed migration of weevils between trees, which changed the functional response of weevils from type II to type III. As a consequence, the proportion of destroyed acorns did not decrease at any level of acorn production, indicating a lowered effectiveness of predator satiation. Furthermore, the effectiveness of the numerical component of the PSH (i.e. the starvation effect of lean years) was also constrained, as indicated by the lack of a relationship between acorn predation and the previous year’s crop size in dense oak patches.

Higher imago abundance following years of higher acorn crop production across all oak densities suggests that weevil population dynamics depend on food (i.e. seed) availability. Yet, the fact that summed crop size in the previous years did not affect seed predation rate in trees growing in dense forest (Fig. 4a) suggests a lack of a starvation effect of predator satiation in these conditions, likely due to two mechanisms. First, microclimate conditions in such habitats compared to those of isolated trees might decrease larval vulnerability to fungal attack or buffer them against summer drought (Menu and Desouhant 2002; Bonal et al. 2015). Second, oaks growing in close proximity allow the relocation of weevils between trees (Fig. 3), which likely adds to the strength of the dependence of imago numbers on the last year’s crop, by increasing the average survival of weevil cohorts (Fig. 4b). In other words, in dense oak patches weevil population decrease in lean years is attenuated by the presence of nearby trees, that are not synchronized with the rest of the population. Therefore, insect abundance is similar across densities, despite twice as low mean acorn availability in dense patches (Table 1). Finally, there is no link between past crop size and acorn predation in oak trees growing in dense patches, due to both numerical and behavioural responses of weevils to higher densities of acorns and oaks.

Despite the fact that effectiveness of predator satiation has long been recognized to potentially depend on dominance or density of focal plants, and has been invoked as an important factor driving the emergence of masting across biomes (Janzen 1971, 1978; Kelly and Sork 2002; Koenig et al. 2003), this notion lacked empirical support. Our work supports the notion that the effectiveness of predation escape decreases with increasing focal plant density, indicating that the intensity of masting should be a function of species dominance or local density attenuated by predator mobility (Ims 1990; Curran and Leighton 2000; Kelly and Sork 2002). It also disproves the notion that relatively immobile, specialist insect seed predators would select for high seeding variability but not synchrony, by showing that synchrony likely matters even for relatively immobile insects like *C. elephas* (Kelly 1994; Kelly and Sork 2002; Koenig et al. 2003). Generally, we would expect species that dominate their communities to show strong masting (as indicated by a high CV_i_ and high synchrony, and thus a high CV_p_), to overcome seed-stage JC effects, and prevent insect re-distribution over proximate conspecifics, that can buffer them against fluctuations in food availability. In contrast, high synchrony and variability should be less important for more interspersed plants, because the negative effects of masting on pre-dispersal seed-feeding insects are stronger the higher the spatial isolation of the tree. Generally, this provides empirical support for the notion that masting and predator satiation should be more important among tree populations that start to dominate their communities or form a continuous forest mass (Kelly et al. 2000; Kelly and Sork 2002), and is consistent with the observation that masting is less frequent and less intense in highly diverse tropical forests (Kelly and Sork 2002; Wright et al. 2005).

Trees growing in denser conspecific patches suffered a higher predation rate, supporting the JC effect at the pre-dispersal stage in our system. On the one hand, the negative density-dependent recruitment is stronger at the seed-to-seedling and the seedling stage than at the seed stage (Comita et al. 2014), which will likely further reduce the regeneration of oaks in dense stands. On the other hand, in masting species, such as oaks, large fruit sets of multiple individuals at one patch can attract seed dispersers (Vander Wall 2001; Gómez 2003; Pesendorfer and Koenig 2016), thus increasing their recruitment likelihood (Herrera et al. 1994; Jansen et al. 2004; Zwolak and Crone 2012; Pesendorfer et al. 2016). Furthermore, in Mediterranean ecosystems, regeneration is strongly shaped by water availability, and older individuals can provide protection from drought (Espelta et al. 1995; Gomez-Aparicio et al. 2008). Therefore, the negative density dependence of predation rate at the pre-dispersal stage could be potentially offset by positive effects at later life stages (Kuijper et al. 2010). Studies examining density dependence at multiple plant life stages will provide important insights into this issue.

To conclude, we found that predator satiation in holm oaks is trumped at high conspecific densities. Up until now, temporal and spatial factors shaping seed predation have been mainly studied separately (Kelly and Sullivan 1997; Yamazaki et al. 2009; Visser et al. 2011; Linhart et al. 2014; Bell and Clark 2016). Our study shows that these processes interact and, depending on the local density of the focal plant, positive or negative density-dependent predation might apply. Therefore, incorporating focal plant density into models could possibly solve the apparently conflicting results in studies of PSH in different plant species, or within the same species across sites (Crone and Lesica 2004; Klinger and Rejmánek 2009; Bell and Clark 2016). Similarly, the lack of negative density dependence in recruitment found in some studies on the JC effect conducted on masting plants (Comita et al. 2014) could be due to the large temporal variation in seed production that may offset the JC effect in some, but not all, years. Finally, the strong influence of plant density on predator satiation effectiveness indicates that community-dominating plants should show high masting intensity, empirically confirming the mechanism often implied to be responsible for the observed variation in masting metrics both within and between species.

## Electronic supplementary material

Below is the link to the electronic supplementary material.

Supplementary material 1 (DOCX 154 kb)
