# Supplementary material for: Effectiveness of predator satiation in masting oaks is negatively affected by conspecific density
Source: Oecologia. 2018 Jan 30;186(4):983–93. doi: 10.1007/s00442-018-4069-7 (PMC5859101; doi:10.1007/s00442-018-4069-7)
Supplement: Supplementary file 1 — Supplementary material 1 (DOCX 154 kb) [file 442_2018_4069_MOESM1_ESM.docx]

Online Supplement 1

Bogdziewicz et al. Is masting enough? Predator satiation suffers from the Janzen-Connell effect. Oecologia.

Figure 1S. The relationship between weevil imago number emerged under the tree and acorn crop in the previous year in dense (triangles) and isolated (circles) patches. Dots represent data points, i.e. the average number of weevil imago trapped per tree in each year. The relationship is insignificant for both densities (negative binomial family GLMM with tree and year included as random effects, and interaction term between crop and density as fixed effects).


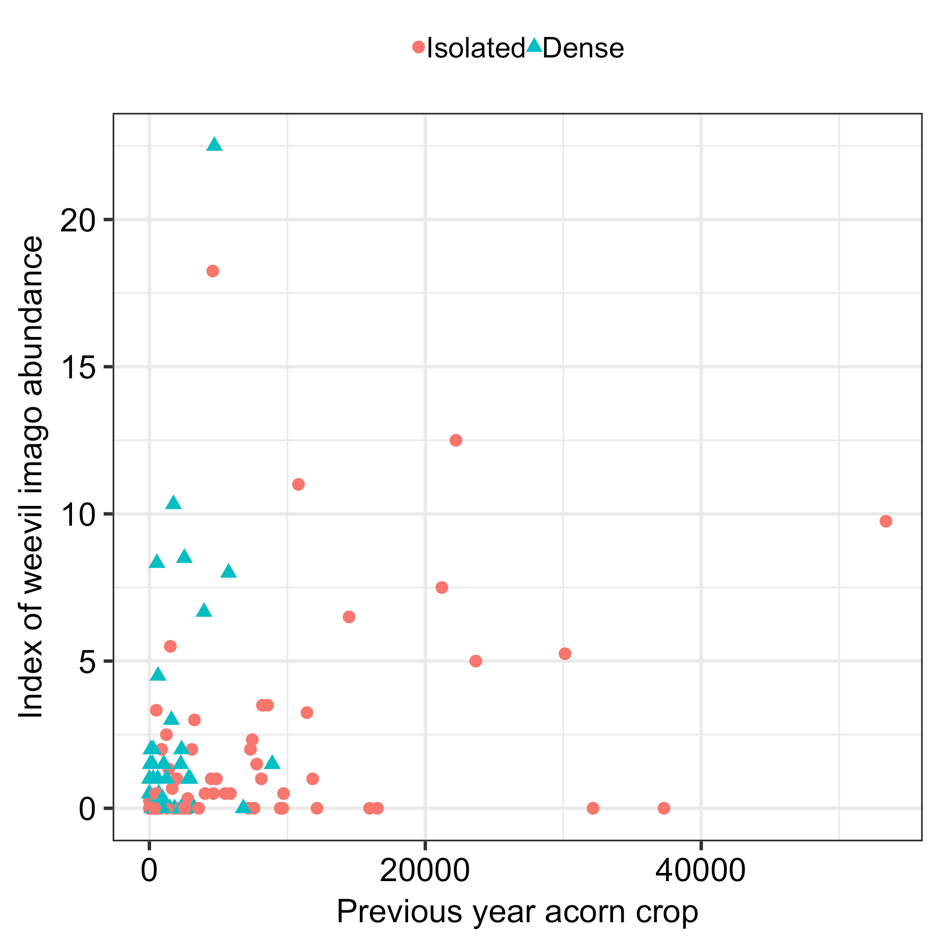


Results of the models fitting with holm oak connectivity included as continuous variable.

Table 1S. Model selection tables. Oak connectivity is included as continuous variable. Models are ranked according to the AICc value; logLik – log-likelihood; AICc – Akaike’s Information Criterion adjusted for small sample size; ΔAICc = AICc_i_ - minAICc; w_i_ – model weight. *×* denotes interaction term.

A) Functional response of weevil predation to acorn abundance in relation to oak connectivity

| Model | D.f. | logLik | AIC | ΔAICc | w_i_ |
| --- | --- | --- | --- | --- | --- |
| Crop size × connectivity + (crop size)^2^ × connectivity | 7 | -1381.9 | 2778.6 | 0 | 1 |
| Crop size × connectivity | 5 | -1445.5 | 2901.4 | 122.77 | 0 |
| Crop size + connectivity | 4 | -1447.6 | 2903.6 | 124.91 | 0 |
| Crop size | 3 | -1449.6 | 2905.5 | 126.83 | 0 |
| Connectivity | 3 | -1498.2 | 3002.7 | 224.03 | 0 |
| Null model | 2 | -1503.7 | 3011.6 | 232.98 | 0 |

The top model includes positive quadratic term of crop size (z = 8.23, p < 0.001), and the negative interaction of crop size with oak connectivity (z = -4.25, p < 0.001). The interpretation is the same as in case of analysis with categorical variable (see the main article for details).

B) Relationship between acorn predation rate and the number of imago emerged under tree in relation to oak connectivity

| Model | D.f. | logLik | AIC | ΔAICc | w_i_ |
| --- | --- | --- | --- | --- | --- |
| Log-adult abundance × connectivity | 5 | -793.2 | 1596.9 | 0 | 1 |
| Log-adult abundance + connectivity | 4 | -814.0 | 1636.4 | 39.49 | 0 |
| Connectivity | 3 | -824.3 | 1655.0 | 58.06 | 0 |
| Null model (intercept only) | 2 | -825.7 | 655.6 | 58.70 | 0 |

The top model includes positive relationship of predation rate with adult abundance (z = 7.27, p < 0.001), and the negative interaction of adult abundance with oak connectivity (z = -6.28, p < 0.001), indicating that the relationship between predation rate and imago abundance diminish with increasing conspecifics connectivity.

C) Relationship between predation rate and summed crop of two previous years in relation to oak connectivity

| Model | D.f. | logLik | AIC | ΔAICc | w_i_ |
| --- | --- | --- | --- | --- | --- |
| Crop size × connectivity | 5 | -1036.8 | 2084.2 | 0 | 1 |
| Crop size + connectivity | 4 | -1058.5 | 2125.3 | 41.16 | 0 |
| Crop size | 3 | -1062.69 | 2131.6 | 47.38 | 0 |
| Connectivity | 3 | -1129.96 | 2266.1 | 181.92 | 0 |
| Null model (intercept only) | 2 | -1132.23 | 2268.6 | 184.37 | 0 |

The top model includes positive effect of the past crop size on predation rate (z = 7.99, p < 0.001), positive effect of oak connectivity on predation (z = 4.54, p < 0.001), and the negative interaction between past crop and oak connectivity (z = -6.21, p < 0.001), indicating that the relationship between past crop size and imago abundance diminish with increasing conspecifics connectivity.

D) Relationship between imago abundance and summed crop of two previous years in relation to oak connectivity

| Model | D.f. | logLik | AIC | ΔAICc | w_i_ |
| --- | --- | --- | --- | --- | --- |
| Crop size × connectivity | 6 | -195.9 | 404.9 | 0 | 0.55 |
| Crop size + connectivity | 7 | -195.4 | 406.2 | 1.34 | 0.28 |
| Crop size | 8 | -195.4 | 408.6 | 3.75 | 0.09 |
| Null model (intercept only) | 5 | -199.3 | 409.4 | 4.56 | 0.06 |
| Connectivity | 6 | -199.2 | 411.6 | 6.74 | 0.02 |

The top model includes positive effect of the past crop size on imago abundance (z = 2.84, p = 0.004), and insignificant effect of oak connectivity on weevil abundance (z = 1.01, p = 0.31). Note that the most parsimonious model is the one with additive effect of crop size and stand density, due to lower number of parameters, and ΔAICc > 2 (Arnold 2000)**.**
